# Studies on the Coordination of Ribosomal Protein Assembly Events Involved in Processing and Stabilization of Yeast Early Large Ribosomal Subunit Precursors

**DOI:** 10.1371/journal.pone.0143768

**Published:** 2015-12-07

**Authors:** Uli Ohmayer, Álvaro Gil-Hernández, Martina Sauert, Pilar Martín-Marcos, Mercedes Tamame, Herbert Tschochner, Joachim Griesenbeck, Philipp Milkereit

**Affiliations:** 1 Lehrstuhl für Biochemie III, Universität Regensburg, Regensburg, Germany; 2 Instituto de Biología Funcional y Genómica (IBFG), CSIC/Universidad de Salamanca, Salamanca, Spain; Univ. of Edinburgh, UNITED KINGDOM

## Abstract

Cellular production of ribosomes involves the formation of highly defined interactions between ribosomal proteins (r-proteins) and ribosomal RNAs (rRNAs). Moreover in eukaryotic cells, efficient ribosome maturation requires the transient association of a large number of ribosome biogenesis factors (RBFs) with newly forming ribosomal subunits. Here, we investigated how r-protein assembly events in the large ribosomal subunit (LSU) rRNA domain II are coordinated with each other and with the association of RBFs in early LSU precursors of the yeast *Saccharomyces cerevisiae*. Specific effects on the pre-ribosomal association of RBFs could be observed in yeast mutants blocked in LSU rRNA domain II assembly. Moreover, formation of a cluster of r-proteins was identified as a downstream event in LSU rRNA domain II assembly. We analyzed in more detail the functional relevance of eukaryote specific bridges established by this r-protein cluster between LSU rRNA domain II and VI and discuss how they can support the stabilization and efficient processing of yeast early LSU precursor RNAs.

## Introduction

Ribosomes play a key role in cellular gene expression by catalyzing the translation of mRNA into proteins. In the unicellular eukaryote *Saccharomyces cerevisiae* (hereafter called yeast), cytoplasmic ribosomes consist of four ribosomal RNAs (rRNA) and 79 ribosomal proteins (r-proteins). These interact with each other in highly defined ways to build the large and the small ribosomal subunit (LSU and SSU, respectively) [1–4]. rRNAs as well as r-proteins contribute to the resulting large ribonucleoprotein complexes being able to translate mRNA according to cellular demands (reviewed in [5,6], see as example also [7]). Three of the four rRNAs are synthesized by RNA polymerase I as part of one precursor transcript (reviewed in [8]). In yeast, as in other eukaryotic cells, RNA polymerase I dependent transcription of rRNA genes takes place in the nucleolus, a sub-compartment of the nucleus. The primary transcript is converted in a timely coordinated series of modification and processing events into mature rRNAs (reviewed in [9,10]). In addition to processing of precursor rRNAs (pre-rRNA), cellular production of ribosomes requires the folding and assembly of their components and the transport of ribosomal precursor particles from the nucleolus to the cytoplasm, the place where mature ribosomes catalyze protein synthesis. In yeast, more than 150 non-ribosomal proteins have been identified, each of which associating with ribosomal precursor particles containing pre-rRNA of defined processing states (reviewed in [11–13]). Yeast mutant analyses indicated that most of these ribosome biogenesis factors (RBFs) somehow stabilize pre-rRNAs and promote specific pre-rRNA processing events and/or nucleocytoplasmic transport of ribosomal precursors. Concomitant with the dynamic association of RBFs with pre-ribosomal particles, assembly of r-proteins with rRNA takes place [12,14]. Accumulating evidence suggests that many of the yeast r-proteins can associate already with the earliest detectable ribosomal pre-rRNAs, although with comparably low binding strength. In the course of pre-ribosomal maturation these weak interactions transform into robust ones as seen in mature ribosomes [15–17]. The timing when interactions with pre-ribosomal particles are stabilized can differ for individual r-proteins (for yeast LSU assembly see [16] and references therein). Thus, groups of yeast r-proteins could be identified with specific assembly properties at early, intermediate or late stages of ribosome maturation. Interestingly, yeast conditional expression mutants of most r-proteins have ribosome maturation phenotypes reminiscent to the ones seen after inactivation or depletion of RBFs: LSU or SSU rRNA precursors are destabilized and specific effects on precursor rRNA processing and/or transport can be observed [14]. Hence, a delay or partial impairment in assembly of most yeast r-proteins seems to perturb pre-rRNA maturation and to trigger substantial elimination of the incomplete ribosomes.

Further insights into how r-proteins mediate stabilization and efficient processing of pre-rRNAs came from recent semi-quantitative proteomic analyses of ribosomal precursor particles isolated from yeast r-protein gene expression mutants [16,18,19]. In pioneering experiments, examples of r-proteins that bind to different LSU domains were chosen to study their impact on LSU assembly. The results of these studies indicated that individual r-proteins can contribute to the efficient recruitment of specific subsets of other r-proteins and can influence the transient interactions of specific RBFs with LSU precursors. Apparently, individual r-protein assembly events, the dynamic association of RBFs, and the stabilization and maturation of pre-rRNA are functionally linked to ensure the efficient production of large quantities of ribosomes of accurate composition.

**Fig 1:**
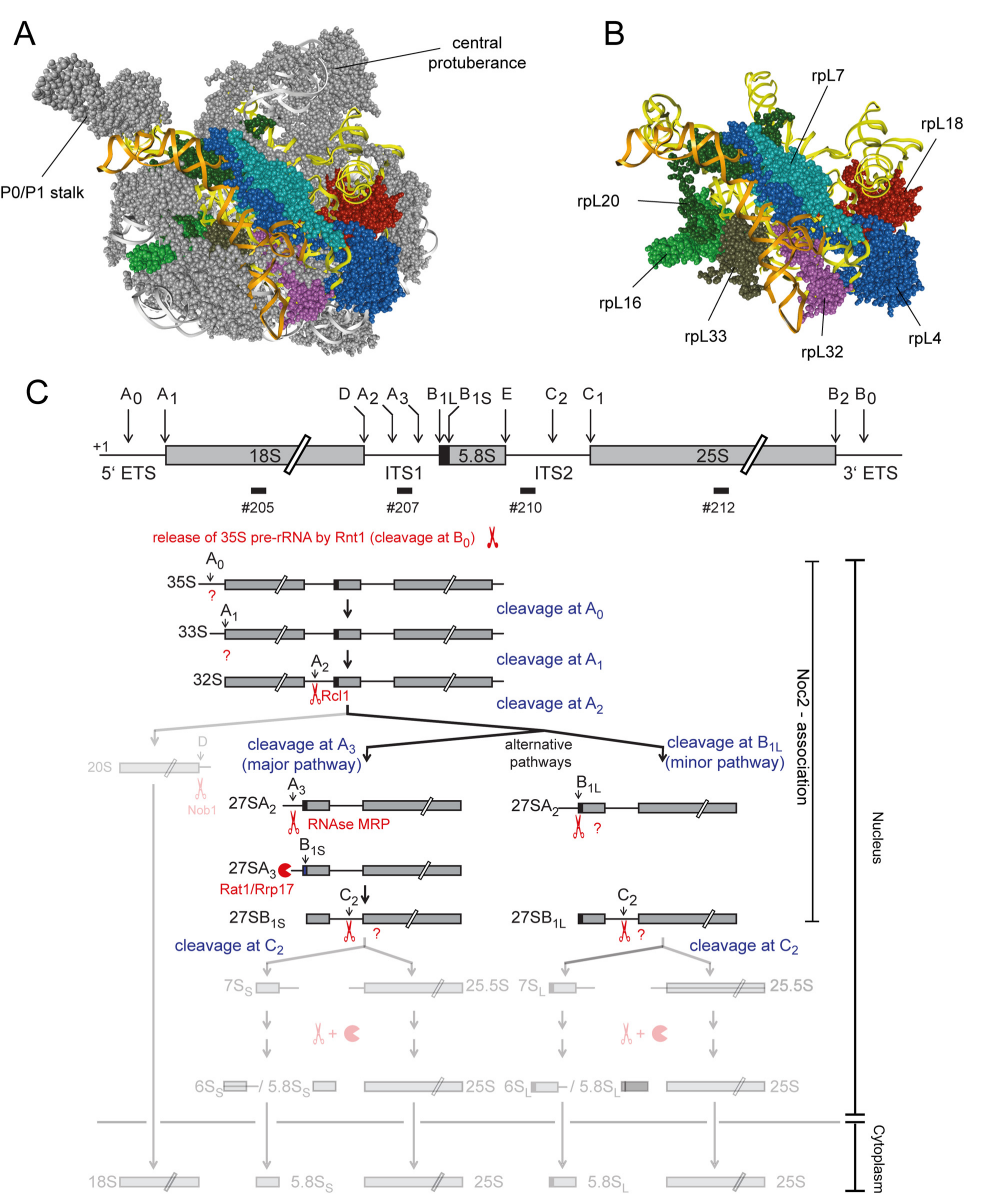
Position of r-proteins studied in this work in ribosomes and overview of early yeast LSU rRNA processing events. The yeast LSU is shown in **A)** and **B)** viewed from the solvent exposed side with LSU rRNA domain II in yellow, LSU rRNA expansion segment ES7 in orange, rpL4/uL4 in dark blue, rpL7/uL30 in light blue, rpL16/uL13 in light green, rpL18/eL18 in red, rpL20/eL20 in dark green, rpL32/eL32 in purple and rpL33/eL33 in brown. Other parts of the LSU are shown in grey in **A)** and are hidden in **B)**. In **C)** is shown an overview of yeast LSU rRNA processing highlighting early events leading to formation of the 5.8S rRNA 5’ end at sites B1S and B1L. The scheme was adapted and modified from [9]. Scissors and pacman symbols illustrate that a pre-rRNA processing event is endonucleolytic or exonucleolytic, respectively. Enzymes catalyzing the respective processing events are indicated, if known, according to [9]. Major pre-rRNAs co-purifying with Noc2 are highlighted on the right.

To further explore these functional connections we aimed to analyze in this work the effects on pre-ribosomal composition of a group of yeast LSU r-proteins binding in a region of the LSU encompassing LSU rRNA secondary structure domain II (nucleotides 652–1455 of yeast 25S rRNA) and the adjacent rRNA expansion segment 7 (ES7, nucleotides 436–624 of yeast 25S rRNA) (see Fig 1A and 1B for binding sites of these proteins in mature ribosomes). The group contained the r-proteins which are named rpL4, rpL7, rpL16, rpL18, rpL20, rpL32 and rpL33 according to the *Saccharomyces cerevisiae* standard protein nomenclature (www.yeastgenome.org and [20]). These r-proteins are named uL4, uL30, uL13, eL18, eL20, eL32 and eL33, respectively, according to the universal r-protein nomenclature recently introduced by Ban *et al*. [21]. Among these proteins, rpL16/uL13, rpL20/eL20 and rpL33/eL33 bridge LSU rRNA domain II with LSU rRNA domain VI, and rpL4/uL4 and rpL32/eL32 form bridges towards LSU rRNA domain I.

Previous work suggested that correct assembly of rpL4 into ribosomes involves, in yeast, its direct interaction with a dedicated chaperone, Acl4 [22,23]. Moreover, other work indicated that depletion of each of the seven selected LSU r-proteins interferes with the earliest steps of yeast LSU pre-rRNA maturation [24–26]: nuclear LSU precursor particles were destabilized after *in vivo* depletion of any of these r-proteins and productive formation of the major 5’ end of 5.8S rRNA, which is called site B1S in yeast (see Fig 1C), was inhibited. Processing at site B1S is thought to be mediated by exonucleolytic trimming of internal transcribed spacer 1 (ITS1) sequences extending 5’ of the 5.8S rRNA region of early LSU pre-rRNAs ([9,10], see Fig 1C). Similar early LSU biogenesis defects were observed in yeast mutants of a few r-proteins interacting with LSU rRNA domain I (5.8S rRNA and nucleotides 1–652 of 25S rRNA, including the ES7 at its 3’ end) and of RBFs with binding sites in LSU rRNA domain I and / or the internal transcribed spacer 2 (ITS2) which emanates from this domain [24,27–30]. Has1 and Pwp1, other factors involved in efficient processing at site B1S, were shown to be required for correct folding of parts of LSU rRNA domain I [31,32]. In addition, mutations in the ITS2 spacer, and in the ES7 region of rRNA interacting with rpL4/uL4, rpL7/uL30, rpL20/eL20 and rpL33/eL33 (see Fig 1B), resulted in perturbed kinetics of the removal of ITS1 spacer sequences extending 5’ of the 5.8S rRNA region of early LSU pre-rRNAs [33,34]. Altogether, these data indicate that the establishment of a defined folding and assembly state within the 5’ region of LSU pre-rRNA is of functional importance for early steps of yeast LSU maturation. In the present work, we analyzed in yeast the impact of seven essential LSU rRNA domain II binding r-proteins on the protein composition of early LSU precursors to further clarify how r-protein assembly events in the LSU rRNA 5’ region are coordinated. Moreover, we addressed how these r-proteins affect the pre-ribosomal association of RBFs that are relevant for initial steps in LSU biogenesis.

## Results

Specific early pre-rRNA maturation phenotypes were previously observed in yeast mutant strains conditionally expressing one of the seven r-proteins rpL4/uL4, rpL7/uL30, rpL16/uL13, rpL18/eL18, rpL20/eL20, rpL32/eL32 and rpL33/eL33 ([24], see introduction). All these proteins are essential for yeast growth and bind to LSU rRNA domain II and/or the neighboring ES7 rRNA region (see Fig 1A and 1B). The yeast strains previously used to characterize the role of the r-proteins for LSU maturation express the respective r-protein under control of the GAL1/10 promoter, which is repressed in medium containing glucose as carbon source. We genetically modified these strains to chromosomally encode a Tandem Affinity Purification (TAP) tagged version of Noc2 enabling affinity purification of pre-ribosomal populations. Noc2 is a LSU biogenesis factor which was shown to associate as part of the Noc2-Noc1-Rrp5 complex and of the Noc2-Noc3 complex with LSU precursor particles of early and intermediate maturation states, respectively ([16,35–37], see Fig 1C for pre-RNAs associated with Noc2p). Noc2 is largely depleted of late nuclear LSU precursor populations for which cleavage at site C2 in the ITS2 pre-rRNA region occurred, and of downstream nuclear and cytoplasmic LSU intermediates [16]. We reasoned that focusing on the characterization of early to intermediate nuclear LSU precursor populations in our analyses should help to reveal specific compositional changes directly related to early pre-rRNA maturation defects observed in the selected r-protein expression mutants. Downstream effects as the failure to recruit late assembling r-proteins and biogenesis factors are expected to be largely masked by this approach. Such downstream effects were previously analyzed by us and by others for some of the r-proteins studied here by characterizing pre-ribosomal particles purified via tagged Rpf2, a factor associating with LSU precursor populations of a wide range of early to late maturation states [18,19].

### Noc2 recruitment into early LSU precursors depleted of r-proteins required for early LSU maturation

**Fig 2:**
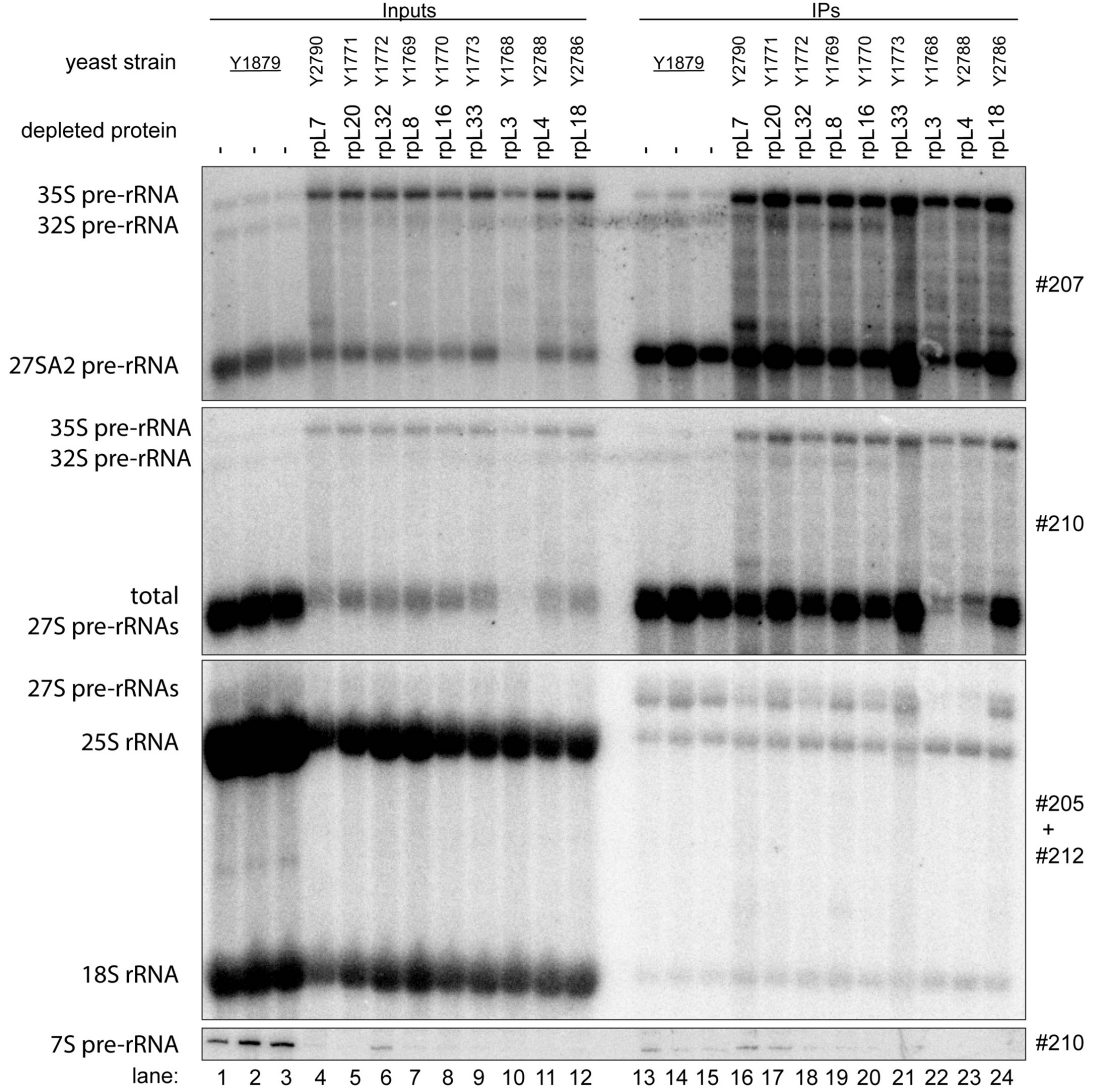
Association of Noc2 with early LSU precursors after *in vivo* depletion of LSU r-proteins required for early LSU pre-rRNA processing. The indicated yeast strains, expressing a chromosomally-encoded TAP-tagged version of the LSU biogenesis factor Noc2, together with either the indicated or no LSU r-protein gene under control of the galactose-inducible GAL1/10 promoter, were cultivated for four hours in glucose-containing medium to shut down expression of the respective LSU r-protein gene. Noc2-TAP and associated pre-ribosomal particles were then affinity purified from the corresponding cellular extracts as described in Materials and Methods. The (pre-) rRNA content of total cellular extracts (“Input” lanes 1–12) or of parts of the affinity purified fractions (“IP”lanes 13–24) was analyzed by northern blotting. (pre-) rRNAs detected by DNA oligonucleotide probes shown on the right are denoted on the left. Equal signal intensities in the Input and IP fractions indicate that 4% of the respective (pre-)rRNA population co-purified with Noc2-TAP.

We first analyzed how *in vivo* depletion of the seven r-proteins affects the association of Noc2 with LSU precursors. To that end, the yeast strains expressing Noc2 in fusion with the TAP tag and one of the selected r-proteins under control of the GAL1/10 promoter were grown in medium containing galactose as carbon source and were then cultivated in glucose containing medium. For comparison, expression mutants of rpL3/uL3 and rpL8/eL8, and a strain not mutated in any r-protein gene were included in these analyses. Previous studies showed that *in vivo* depletion of any of these two r-proteins leads to similar early defects in LSU pre-rRNA maturation as observed in case of the seven LSU rRNA domain II / ES7 binders [24,38]. After cultivation for four hours in glucose containing medium, cells were harvested and total cellular extracts were prepared. RNA was isolated from a fraction of the extracts and pre-rRNA processing phenotypes were analyzed by northern blotting (Fig 2, lanes 1–12). Three major differences between pre-rRNA patterns in the mutant and control cells further corroborated the previously reported early pre-rRNA processing phenotypes in all analyzed LSU assembly mutants: a strong decrease in levels of 7S pre-rRNA (Fig 2, compare 7S pre-rRNA levels in lanes 1–3 with lanes 4–12), a clear accumulation of 35S pre-rRNA (Fig 2, compare 35S pre-rRNA levels in lanes 1–3 with lanes 4–12), and a clear increase in the proportion of 27S pre-rRNAs with 5’ ends extending to site A2 (Fig 2, compare ratio of 27SA2 pre-rRNA to total 27S pre-rRNA in control lanes 1–3 with lanes 4–12). A pronounced destabilization of early and intermediate LSU precursor RNAs was observed after *in vivo* depletion of rpL3/eL3 (Fig 2, compare levels of total 27S pre-rRNA in lane 10 with lanes 4–9 and 11–12).

Residual yeast cellular extracts were used to affinity purify Noc2-TAP on magnetic beads coated with rabbit immunoglobuline G (IgG). One part of the washed beads was subjected to proteomic analyses (see below), whereas the other part was used for RNA extraction followed by northern blotting experiments. The latter analyses indicated that early and intermediate pre-RNAs were co-purifying with Noc2-TAP from extracts of most of the mutant cells with similar efficiency when compared to co-purification from extracts of control cells (Fig 2, compare levels of 27SA2 pre-rRNA and total 27S pre-rRNA in input lanes 1–12 and eluate lanes 13–24). Reduced co-purification of 27S pre-rRNAs with Noc2-TAP was observed after *in vivo* depletion of rpL4/uL4 (Fig 2, compare levels of total 27S pre-rRNAs in input lane 11 and eluate lane 23), pointing to a partially impaired Noc2 association with some LSU precursor populations in this situation. The low amounts of 27S pre-rRNA co-purifying with Noc2-TAP after *in vivo* depletion of rpL3/uL3 reflected the pronounced destabilization of these precursors observed in this strain (Fig 2, total 27S pre-rRNA levels in lane 10 and 22). In conclusion, the data indicated that none of the tested r-proteins implicated in early steps of rRNA maturation was strictly required for recruitment of Noc2p into pre-ribosomal particles.

### Impact of r-proteins required for early LSU maturation on the association of RBFs with early LSU precursors

**Fig 3:**
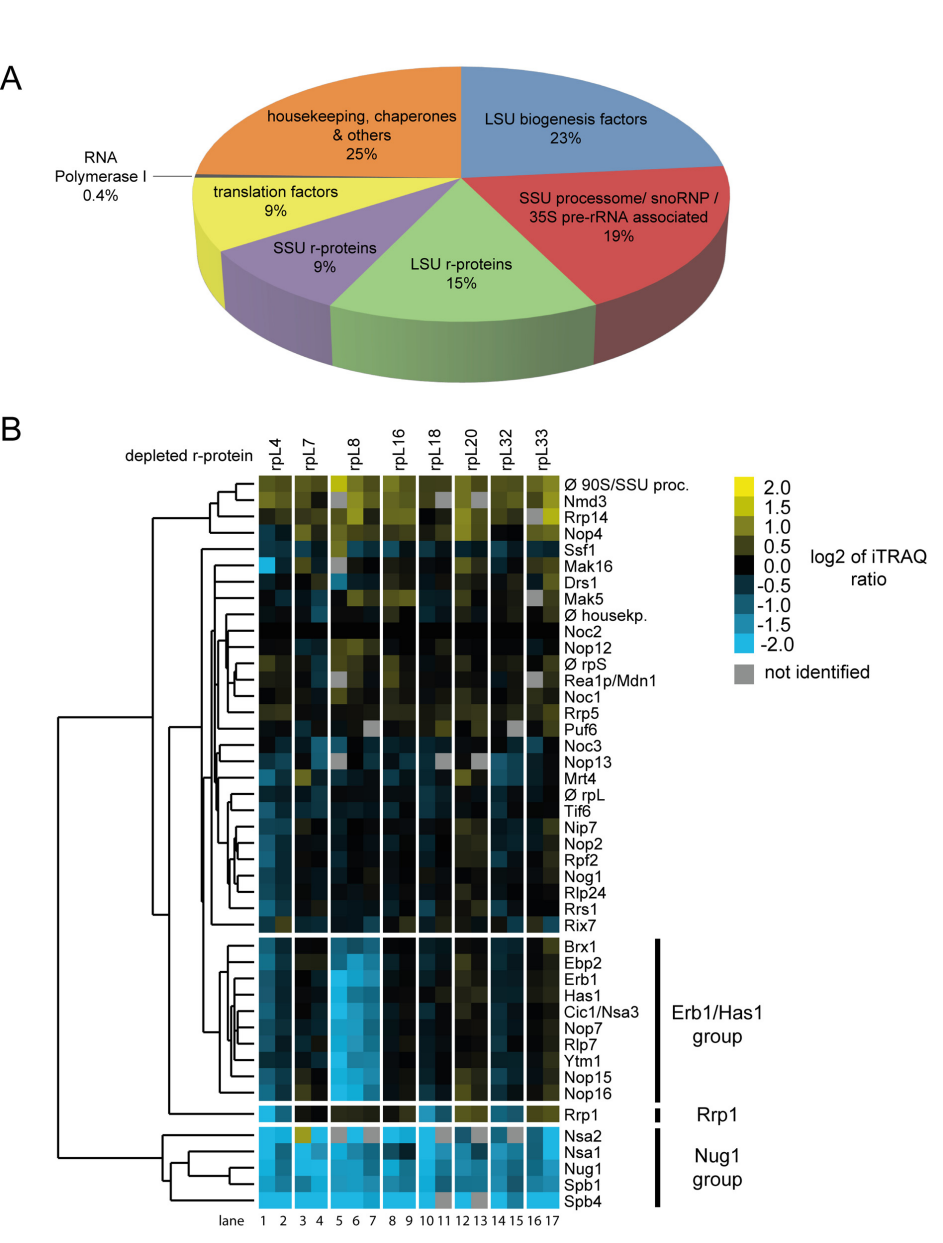
Changes in the association of LSU biogenesis factors with early LSU precursors after *in vivo* depletion of LSU r-proteins required for early LSU pre-rRNA processing. Conditional r-protein expression mutants or wild type cells were cultivated as described in Fig 2. Noc2-TAP and associated pre-ribosomal particles were then affinity purified from corresponding cellular extracts (see Materials and Methods). Proteins in Noc2-TAP fractions were identified and quantified by mass spectrometry. Isobaric labeling of peptides (iTRAQ, see Materials and Methods) was used to directly compare levels of individual proteins in Noc2-TAP fractions from wild type cells with the respective levels in Noc2-TAP fractions from conditional r-protein expression mutants. In **A)** is shown the average proteome composition of nine Noc2-TAP fractions analyzed in this study by mass spectrometry. In **B)** the changes in levels of individual LSU biogenesis factors in Noc2-TAP fractions from mutant versus wild type cells as determined by iTRAQ analyzes are summarized. Each pair wise comparison (“mutant *versus* wild type”) was performed at least twice starting from independent cell cultures. Data from 17 pairwise comparisons were used to detect co-behaving groups of LSU biogenesis factors by a clustering algorithm (see Materials and Methods). Similar behaving proteins are grouped in the same branch of the dendrogram depicted on the left. The iTRAQ ratios of LSU biogenesis factors for each individual pairwise comparison are shown as heat map using the color code depicted on the upper right. Only LSU biogenesis factors were included in these analyses, which were identified in at least 70% of the 17 pairwise comparisons. Groups of LSU biogenesis factors mentioned in the text are highlighted by bars on the right.

The efficient purification via Noc2-TAP of residual early and intermediate pre-ribosomal populations accumulating in the mutant strains allowed us to study their protein composition in further detail. Noc2-TAP fractions from control cells, from expression mutants of the seven LSU rRNA-domain II / ES7 binders and, for comparison, of the LSU rRNA domain I binder rpL8/eL8, were included in these analyses. Proteins eluting from the beads were digested with trypsin and the resulting peptides were identified by mass spectrometry (MS). Moreover, peptides derived from the various purifications were labeled with differential isobaric tags (iTRAQ reagents, see Materials and Methods) enabling pairwise comparison of individual protein levels in pre-ribosomal preparations from mutant and control cells. The composition of the proteome identified in the Noc2-TAP fractions reflected well the pre-rRNAs co-purifying with Noc2-TAP: a major portion of the identified peptides belonged to LSU biogenesis factors associated with early and intermediate LSU precursors (Fig 3A). In addition, significant amounts of peptides of SSU processome components could be detected [39]. Consistently, these proteins were described to be recruited to the earliest detectable pre-ribosomal populations containing 35S pre-rRNA, the common precursor to both SSU and LSU rRNA (see Fig 1C). Thus, the high amount of 35S pre-rRNA associated with Noc2-TAP (Fig 2, 35S pre-rRNA levels in lanes 16–24) matched with the identification of many SSU processome components in Noc2-TAP fractions. The detection of peptides derived from RNA polymerase I suggested that a portion of the purified pre-ribosomal populations consisted of co-transcriptionally formed ribonucleoprotein assemblies [36]. Besides, peptides belonging to general housekeeping enzymes, chaperones and translation factors were identified which probably reflected non-specific background also indicated by the presence of some amounts of mature 18S and 25S rRNAs in the Noc2-TAP fractions (Fig 2, 25S rRNA and 18S rRNA levels in lanes 13–24).

The semi-quantitative proteomic approach chosen for characterization of the Noc2-TAP fractions allowed for detection of significant changes in the respective protein compositions. First, changes in levels of the LSU biogenesis factors identified in these experiments were analyzed. For all eight r-protein expression mutants included in these experiments the levels of LSU biogenesis factors found in the Noc2-TAP fraction were compared with those found in Noc2-TAP fractions purified from control cells. Each pairwise comparison was performed on at least two biological replicates. The data were normalized for the respective differences in the levels of the bait protein Noc2-TAP and they were visualized as heat maps (S1 Fig). Hierarchical clustering analyses using datasets of 17 pairwise comparisons between LSU precursor populations isolated from r-protein expression mutants and control cells helped to reveal significant similarities and differences in their LSU biogenesis factor composition (Fig 3B). For all r-protein expression mutants analyzed a group of LSU biogenesis factors, including Nug1 (Fig 3B, factors marked as Nug1-group), was observed to be strongly depleted from Noc2-TAP associated pre-ribosomes. Previous studies indicated that several of these RBFs are mainly involved in pre-rRNA processing steps downstream of the ones affected in the r-protein expression mutants analyzed here [40–46]. Among the Nug1-group proteins, Nsa2 was previously shown to directly interact with rRNA helices 42, 89 and 90, located in LSU rRNA domains II and V, at the ribosomal subunit interface region [47]. Another Nug1-group protein, Spb1, is a methyltransferase involved in methylation of a guanine residue very close to the Nsa2 interaction sites at the subunit interface region [44]. Thus, r-protein assembly events in LSU rRNA domain I and II seem to impact the association of downstream acting ribosome biogenesis factors with interaction sites in LSU rRNA domain II and V at the subunit interface region. These results were in support of presumable hierarchical effects of ribosomal assembly events at the subunit surface in LSU rRNA domains I and II on downstream maturation events in the subunit interface region [19]. A clear decrease in the levels of many early acting LSU biogenesis factors was detected in Noc2-TAP associated pre-ribosomal populations depleted of LSU rRNA domain I binding r-protein rpL8/eL8 (Fig 3B, lanes 5–7, levels of factors marked as Erb1/Has1 group) [28,32,48–50]. Interestingly, association of these proteins, termed here “Erb1/Has1 group”, with pre-ribosomes was clearly less affected after expression shut down of any of the seven LSU rRNA domain II / ES7 binding r-proteins (Fig 3B compare levels of Erb1/Has1 group factors in lanes 5–7 with levels in lanes 1–4 and 8–17). These observations were in agreement with the previously reported differential impact of rpL8/eL8 and rpL7/uL30 on LSU precursor association of Erb1/Has1 group proteins [18]. Effects on another early acting LSU biogenesis factor, Rrp1, significantly differed between expression mutants of the seven LSU rRNA domain II / ES7 binders (Fig 3B) [51]. Reduced levels of Rrp1 in Noc2-TAP fraction were reproducibly seen after *in vivo* depletion of rpL4/uL4, rpL18/eL18 or rpL32/eL32. In contrast, *in vivo* depletion of the other analyzed LSU rRNA domain II / E7 binders (rpL7/uL30, rpL16/uL13, rpL20/eL20 and rpL33/eL33) or of the LSU rRNA domain I binder rpL8/eL8 led to a slight increase of Rrp1 levels in the purified pre-ribosomes. The globular domains of rpL4, rpL18 and rpL32 all bind to a sub-region of LSU rRNA domain II distinct from the binding sites of the other analyzed r-proteins (see Fig 1A and 1B). Pre-ribosomal association of the early acting LSU biogenesis factor Rrp1 might therefore depend on the local structure formed by rpL4, rpL18 and rpL32. In summary, these results indicated that yeast r-proteins involved in the first steps of LSU pre-rRNA processing have a significant impact on the association of sub-groups of RBFs with early LSU precursors. Moreover, different binding sites of these r-proteins in the 5’ region of LSU rRNA correlated with specific effects on early acting LSU biogenesis factors as Rrp1 and the Erb1/Has1 group factors.

### Impact of r-proteins required for early LSU maturation on r-protein assembly states of early LSU precursors

**Fig 4:**
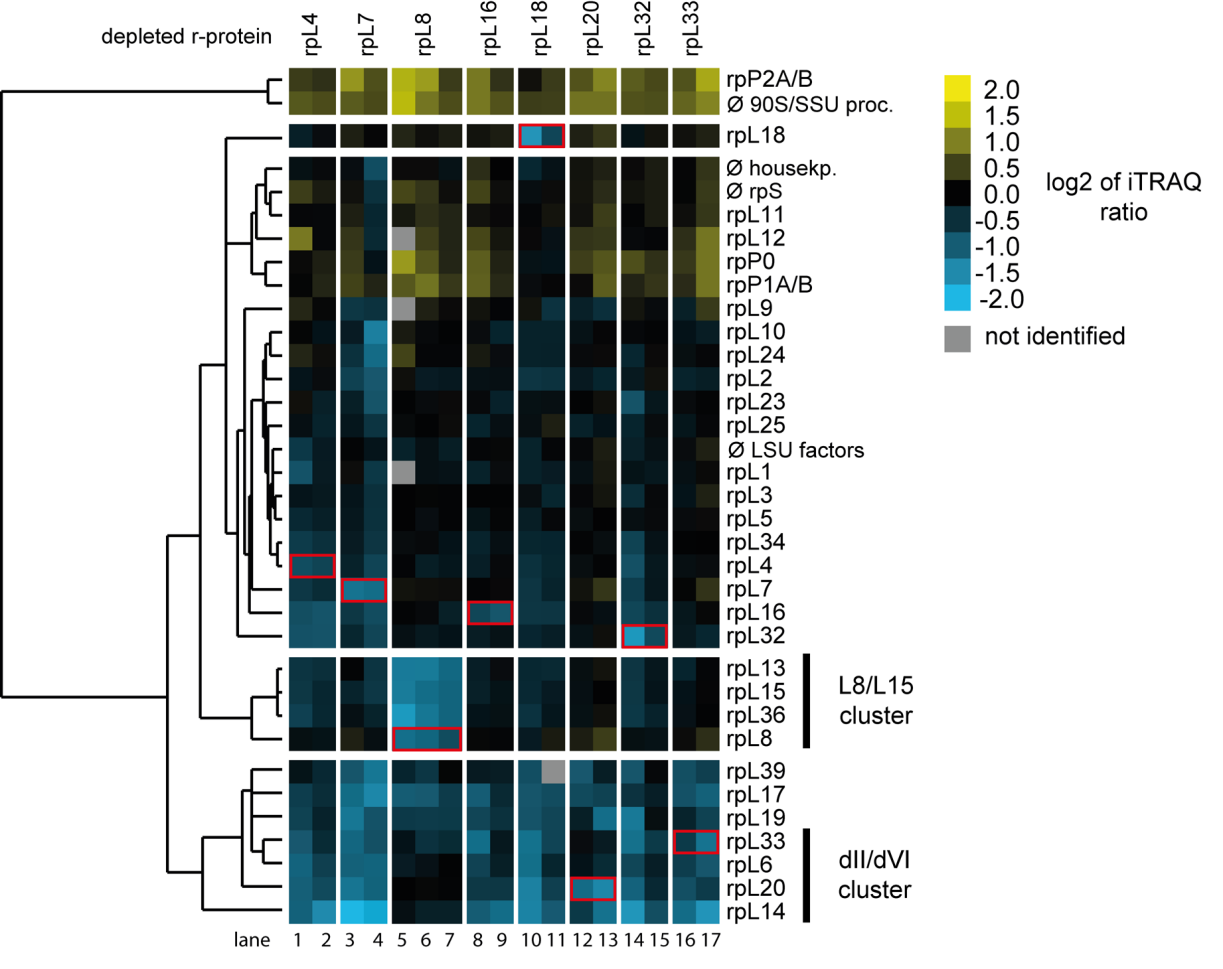
Changes in r-protein assembly states of early LSU precursors after *in vivo* depletion of LSU r-proteins required for early LSU pre-rRNA processing. The dataset generated as described in Fig 3 was analyzed in respect to changes in r-protein levels in Noc2-TAP fractions isolated from wild type cells or from r-protein expression mutants. Observed changes in levels of r-proteins and the results of clustering analyses are visualized as described in Fig 3B. Groups of r-proteins mentioned in the text are highlighted by bars on the right. Only r-proteins which were identified in at least 70% of the 17 pairwise comparisons were included in these analyses. The r-proteins whose expression was shut down in the respective experiment (“mutant *versus* wild type”) are highlighted by a red box.

**Fig 5:**
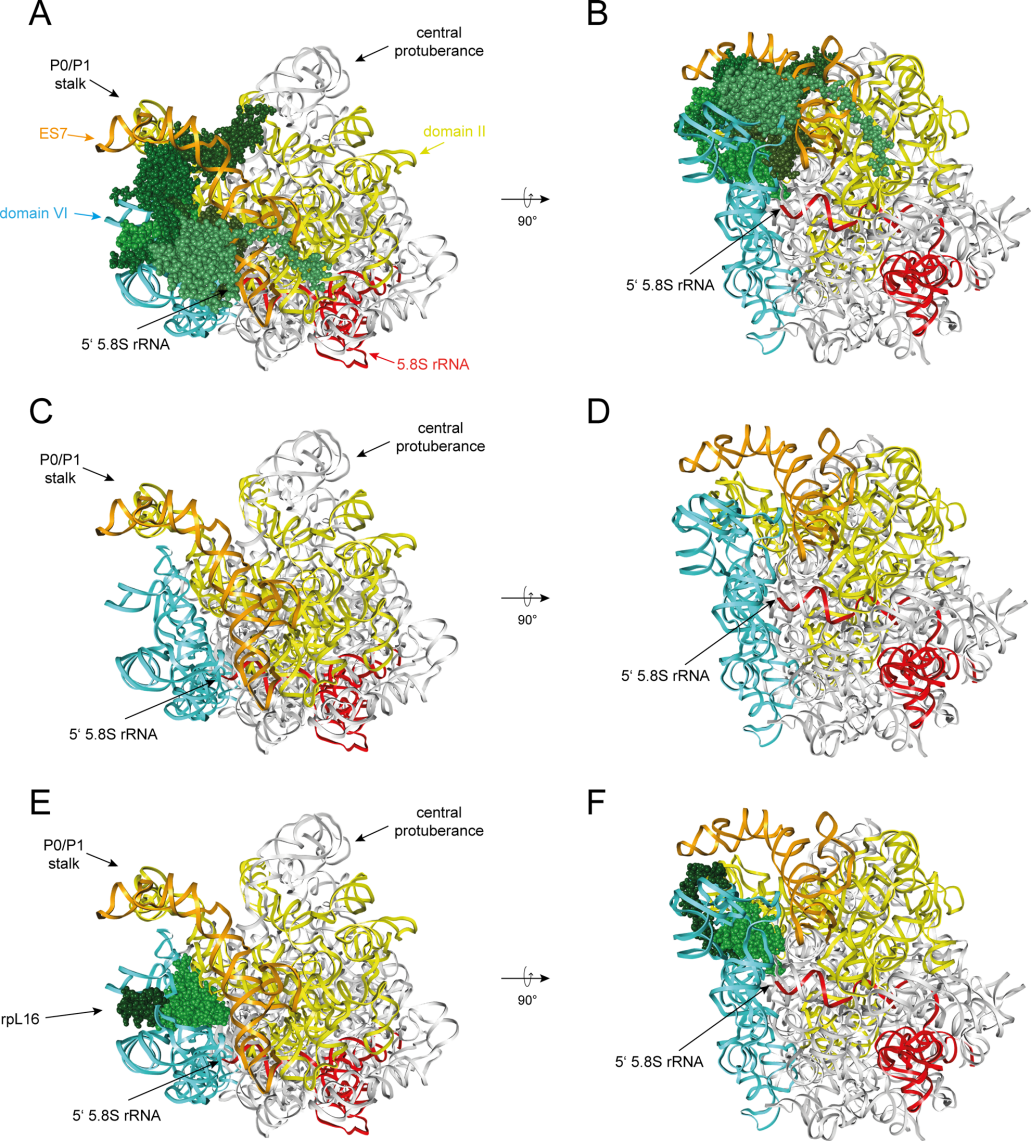
Interactions of dII/dVI cluster r-proteins with rRNA in mature ribosomes. The yeast LSU is shown as in Fig 1 viewed from the solvent exposed side in **A)**, **C)** and **E)**. It is rotated by approximately 90 degree around a horizontal axis in **B)**, **D)** and **F)**. In **A)**–**F)** LSU rRNA domain II is colored in yellow, LSU rRNA expansion segment 7 in orange, LSU rRNA domain VI in blue, 5.8S rRNA in red and other parts of the LSU rRNA in grey. In **A)** and **B)** dII/dVI cluster r-proteins are the only r-proteins shown and colored in shades of green. In **C)** and **D)** only rRNA is visualized and in **E)** and **F)** rpL16/uL13 is the only r-protein shown, with its globular domain in light green and its C-terminal clamp-like domain in dark green.

Next, the semi-quantitative proteomic dataset described above was analyzed in regard to specific LSU r-protein assembly phenotypes. For this, changes in levels of all detected LSU r-proteins in the 17 experiments were, again, first visualized as heat maps (S2 Fig). Hierarchical clustering analyses were then performed to detect significant differences in LSU r-protein levels (Fig 4). Importantly, these analyses indicated that *in vivo* depletion of any of the eight r-proteins consistently diminished the respective r-protein in Noc2-TAP associated pre-ribosomes (see Fig 4 and S2 Fig, levels of *in vivo* depleted r-proteins highlighted by red boxes). This result strengthened the conclusion that the chosen experimental conditions allowed us to detect r-protein assembly phenotypes in early LSU precursor particles although background level of mature ribosomes were co-purified with Noc2-TAP (see above, 25S rRNA and 18S rRNA levels in Fig 2, lanes 13–24). Indeed, a clear effect on the assembly of the three r-proteins rpL13/eL13, rpL15/eL15 and rpL36/eL36 into Noc2-TAP particles was observed after *in vivo* depletion of LSU rRNA domain I binder rpL8 (see Fig 4, r-proteins marked as L8/L15 cluster, lanes 5–7). These proteins together with rpL8/eL8 form one protein cluster associated with LSU rRNA domain I. Assembly of the L8/L15 cluster was not significantly affected after depletion of any of the seven LSU rRNA domain II / ES7 binding r-proteins. This result agreed well with previous results for the expression mutant of rpL7/uL30 [18]. On the other hand, levels of a common group of r-proteins specifically dropped in Noc2-TAP associated pre-ribosomes after expression shut down of any of the seven LSU rRNA domain II / ES7 binding r-proteins (see Fig 4, r-proteins rpL39/eL39, rpL17/uL22, rpL19/eL19, rpL33/eL33, rpL6/eL6, rpL20/eL20 and rpL14/eL14). Strikingly, four of these proteins (rpL33/eL33, rpL6/eL6, rpL20/eL20 and rpL14/eL14) form together with rpL16/uL13 one r-protein cluster in LSU rRNA domain II establishing extensive contacts with ES7, LSU rRNA domain VI (25S rRNA nucleotides 2996–3397) and ES39 (25S rRNA nucleotides 3152–3295) (see Fig 5A–5D for location of these r-proteins in mature ribosomes). ES39 is a eukaryote specific expansion segment emanating from LSU rRNA domain VI. The group of these four r-proteins together with rpL16/uL13 will be termed hereafter “dII/dVI r-protein cluster”. Numerous direct interactions between dII/dVI cluster r-proteins in mature ribosomes (see Fig 5A and 5B) provide a likely explanation why these r-proteins were often found to be co-depleted, with rpL16/uL13 playing a more upstream role in cluster formation. The observation that *in vivo* depletion of LSU rRNA domain II binders rpL4, rpL7, rpL18 and rpL32, which themselves are not part of the dII/dVI cluster, affected its assembly (Fig 4) argued for dII/dVI cluster formation being a downstream event in LSU rRNA domain II assembly. As shown for rpL33/eL33, rpL20/eL20, rpL16/uL13 and rpL6/eL6 (see Fig 2, [19,24,26]), at least four of the five dII/dVI cluster r-proteins are individually required for the same early pre-rRNA maturation step generating the major 5.8S rRNA 5’ end at site B1S. In sum, these results indicated that formation of the dII/dVI r-protein cluster is a downstream event in the yeast LSU rRNA domain II assembly pathway important for early steps of LSU rRNA processing.

### Role of the C-terminal extension of rpL16 for early LSU maturation

**Fig 6:**
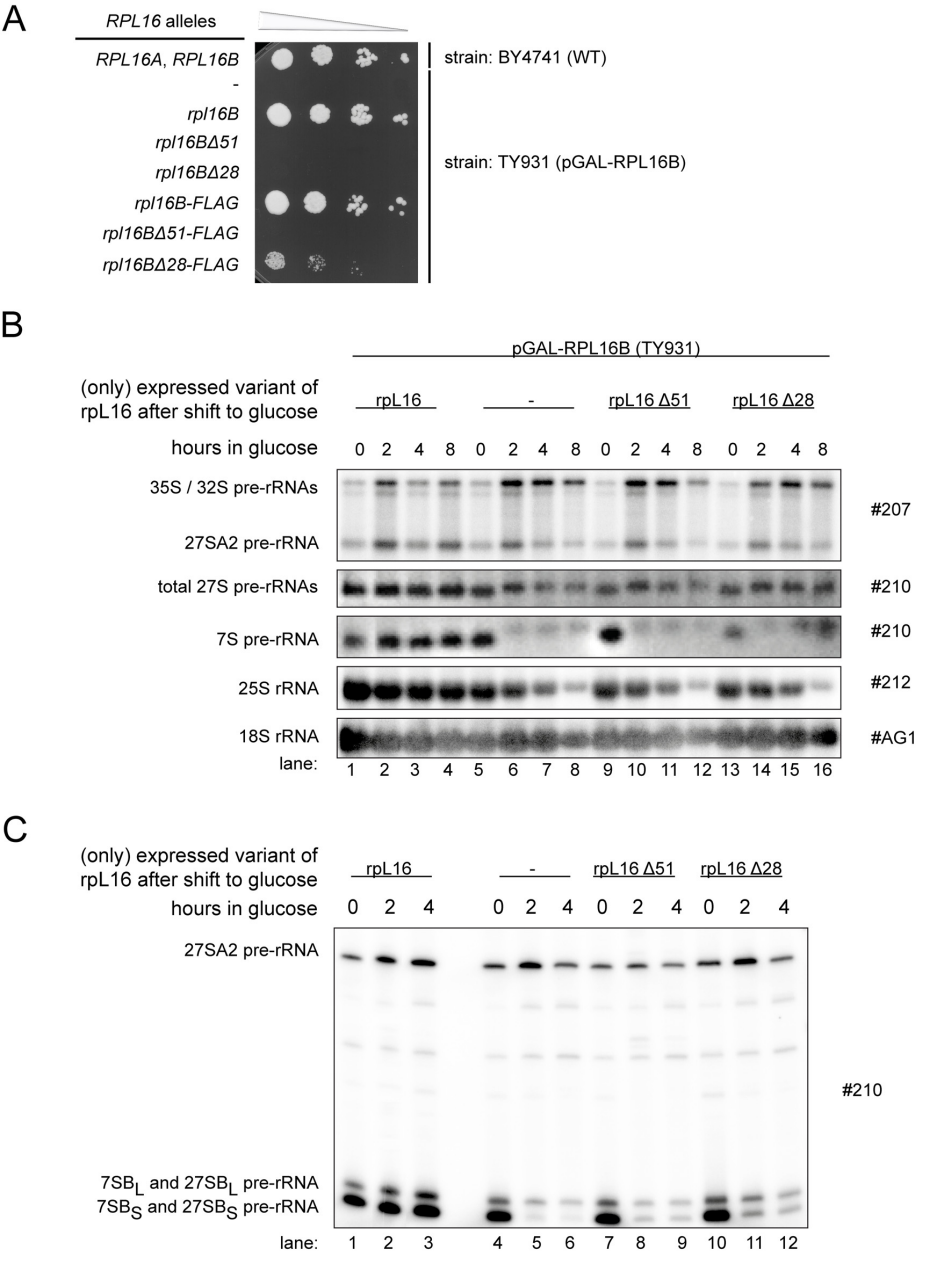
Impact of the C-terminal clamp-like domain of rpL16 on yeast growth and LSU rRNA processing. **A)** The wild type (WT) *RPL16A*, *RPL16B* yeast strain BY4741 and transformants of yeast strain TY931 expressing the full length allele of *RPL16B* under control of the GAL1/10 promoter (*pGAL-RPL16B*), and containing the indicated *RPL16B*alleles on low copy (lc) plasmids or the FLAG-tagged versions of *RPL16B* alleles on high copy (hc) plasmids, were grown in selective complete medium with galactose as carbon source (SCGal-U) to an OD_600_ of 0.6 at 30°C. The same amount of cells of each culture was spotted on solid YPD medium in a 1:10 serial dilution series and incubated for 3 days at 30°C. **B)** and **C)** strains from **A)** harbouring the lc plasmids coding for the indicated truncated protein variants of rpL16B were grown at 28°C in SCGal-U and then cultivated in glucose containing medium (YPD). Total RNA was extracted from cells at the indicated time points, and steady-state levels of pre-rRNAs were assayed by northern blotting **B)**, or primer extension and denaturing gel electrophoresis **C)**.

An obvious feature of the dII/dVI r-protein cluster is the extensive contact interface it establishes between LSU rRNA domain II, ES7 and LSU rRNA domain VI, including ES39, in mature ribosomes. We aimed to test whether these protein bridges between domain II and VI are of functional importance for yeast pre-rRNA processing and/or stabilization. The latter option was especially attractive since examination of the LSU structure suggests that anchoring of LSU rRNA domain VI to domain II and ES7 reinforces a folding state in mature ribosomes likely hindering the access of exonucleases to the 5' end of 5.8S rRNA. In fact, stabilized by the dII/dVI r-protein cluster, large parts of LSU rRNA domain VI, including ES39, cover in a “lid-like” configuration the 5’ end of 5.8S rRNA (see Fig 5A–5D). DII/dVI cluster protein rpL16/uL13 seems to play a prominent role in anchoring ES39 and LSU rRNA domain VI on domain II. Its globular domain is sandwiched between LSU rRNA domain II and helices 97 and ES39 of LSU rRNA domain VI. The eukaryote specific C-terminal extension of rpL16/uL13 folds in a clamp-like way, stabilized by interactions with the C-terminal domain of rpL14/eL14, around ES39 (see Fig 5E–5F, see discussion in [52] in regard to evolutionary conservation of rpL16/uL13). We constructed two plasmids supporting constitutive yeast expression of rpL16/uL13 variants with either partial (rpL16Δ28) or full (rpL16Δ51) truncation of the clamp-like domain. These plasmids, together with an empty vector and a plasmid coding for full-length rpL16/uL13, were transformed into yeast strain TY931, in which expression of rpL16/uL13 is under control of the conditional GAL1/10 promoter. Growth tests on glucose containing plates indicated that truncation of the rpL16 clamp-like domain severely interfered with essential cellular functions of yeast rpL16/uL13 (Fig 6A). To analyze possible resulting pre-rRNA processing phenotypes, the respective cells were grown in galactose containing medium and expression of full length rpL16/uL13 was shut down by cultivation in glucose containing medium for up to 8 hours. Samples were taken at intermediate times and total cellular RNA was extracted and analyzed by northern blotting and primer extension reactions. The results of these experiments indicated that the rpL16 clamp-like domain is required for efficient trimming of ITS1 pre-rRNA regions extending from site B1S, and for stabilization of LSU precursors. Thus, the ratio of 25S rRNA to 18S rRNA decreased over time in cells expressing only the truncated variants of rpL16/uL13 (Fig 6B, compare 25S rRNA to 18S rRNA ratios in lanes 1–4 with ratios in lanes 9–12 and 13–16). Furthermore, after 2 hours shift to glucose containing medium levels of 7S pre-rRNA were significantly decreased (Fig 6B, compare 7S pre-rRNA levels in lane 2 with levels in lane 10 and lane 14), and the proportion of 27S pre-rRNAs with 5’ ends extending to site A2 was increased (Fig 6B, compare 27SA2 pre-rRNA to 27S pre-rRNA ratios in lane 2 with ratios in lanes 10 and 14). Finally, the ratio of 27SBS to 27SBL pre-rRNA was strongly diminished (Fig 6C, compare 27SBL to 27SBS ratios in lane 2 with ratios in lanes 8 and 11). The observed pre-rRNA processing phenotype in the cells expressing no rpL16/uL13 was slightly more pronounced compared to the phenotype in cells expressing the two rpL16 clamp-truncation variants (Fig 6B, lanes 5–8 and Fig 6C, lanes 4–6, compare also with [24]).

**Fig 7:**
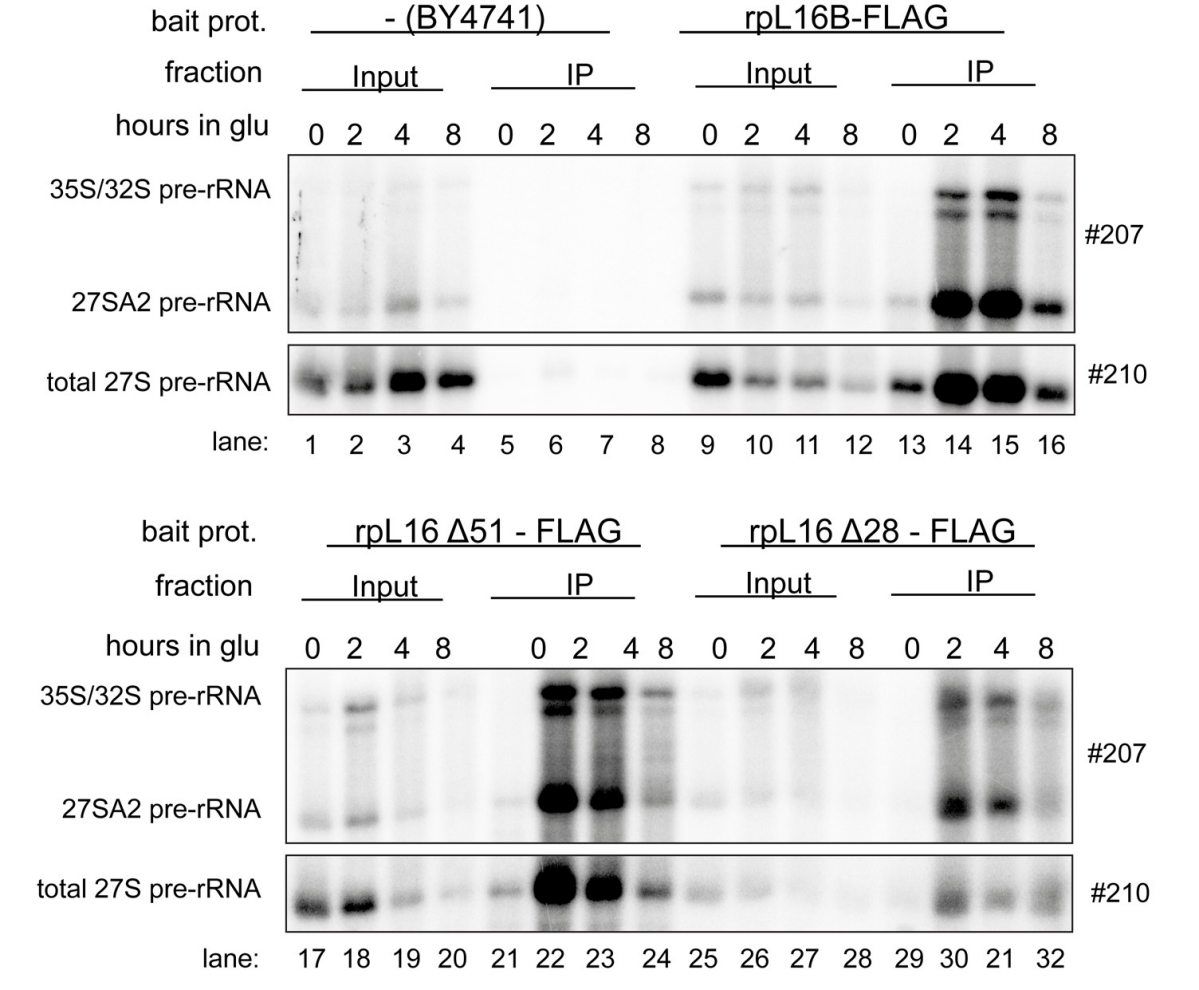
Impact of the C-terminal clamp-like domain of rpL16/uL13 on recruitment into early yeast LSU precursors. Yeast strains Hm653, Hm654 and Hm655 ectopically expressing only the indicated FLAG tagged variants of rpL16/uL13 were grown in selective complete medium with galactose as carbon source and were then cultivated for the indicated times in glucose containing medium. Cells were harvested and subjected to affinity purification using an anti-FLAG matrix as described in Materials and Methods. Wild type yeast strain BY4741 expressing only untagged rpL16/uL13 was included as control in these analyses. The (pre-) rRNA content of the total cellular extracts (“Input” lanes) or of parts of the affinity purified fractions (“IP” lanes) were analyzed by northern blotting using the indicated probes.

These defects in early steps of LSU maturation might be due to a failure of the truncated forms of rpL16/uL13 to assemble into LSU precursors. Alternatively, the rpL16 clamp-like domain might have a more direct, stabilizing function in early LSU maturation, after the recruitment of the protein into pre-ribosomes. To distinguish between these options, we used cells expressing FLAG tagged variants of rpL16/uL13 to test directly the association of the truncated rpL16/uL13 variants with early LSU precursors failing to mature in this situation. Total cellular extracts were prepared and incubated with sepharose beads coated with antibodies recognizing the FLAG-tag expressed in fusion with the tested variants of rpL16. After washing of the beads, RNA co-purifying with the tagged variants of rpL16 was isolated and analyzed by northern blotting. The results of these experiments indicated that similar to full length rpL16/uL13, both truncated versions of rpL16/uL13 assembled into early LSU precursors containing 27SA2 pre-rRNA (Fig 7, 27SA2 and total 27S pre-rRNA levels in IP lanes 22–24 and 30–32). In sum, these data argued against a strict requirement of the C-terminal part of yeast uL13/rpL16 for its recruitment into pre-ribosomes. They rather favored a possible direct role of the clamp-like domain in stabilizing early LSU precursors and rendering them competent for efficient exonucleolytic trimming of ITS1 pre-rRNA sequences.

## Discussion

In the present work we identified formation of an r-protein cluster at the interface of LSU rRNA domain II, ES7 and LSU rRNA domain VI (“dII/dVI cluster”) as a downstream event in the yeast LSU rRNA domain II assembly pathway important for early steps of LSU rRNA processing. Previous studies indicated that a group of several LSU biogenesis factors, including Mak5, Rpf1, Nop16 and Ebp2 are genetically linked with each other and with the dII/dVI cluster r-protein rpL14 [52]. The data obtained here did not provide evidence for significantly decreased recruitment of Mak5 or its genetic interactors into pre-ribosomes upon failure of dII/dVI r-protein cluster formation (Fig 3B, S1 Fig). We could neither detect significant alterations in the recruitment of many other early acting RBFs, as for example proteins belonging to the Erb1/Has1 group. One exception was Pwp1, a non-essential RBF required for efficient LSU rRNA domain I folding and early trimming of ITS1 pre-rRNA regions [31]. Quantification of very few Pwp1 peptides in some of the pre-ribosomal purifications indicated its depletion when LSU rRNA domain II assembly was impaired (S1 Fig). Surprisingly, Pwp1 was described to be important for stable association of many of the Erb1/Has1 group of proteins [31] which we found not significantly affected in the LSU rRNA domain II assembly mutants analyzed here. Thus, it seems possible that Pwp1 dissociates from LSU precursors with failure in dII/dVI cluster formation after promoting recruitment of the Erb1/Has1 group of LSU biogenesis factors. Apart from that, the three r-proteins rpL39/eL39, rpL17/uL22 and rpL19/eL19 (Fig 4) were often found co-depleted with dII/dVI cluster r-proteins from misassembled LSU precursors (Figs 3B and 4). Incomplete formation of the dII/dVI r-protein cluster might directly affect assembly of rpL17/uL22 and rpL19/eL19 since both have ribosomal interaction sites in LSU rRNA domains II and VI. Previous yeast mutant analyses implicated these r-proteins primarily in downstream events of LSU pre-rRNA maturation. Thus, no specific effects on early exonucleolytic trimming at site B1S, as indicated by changes in ratios of 27B1S to 27B1L rRNA seen upon failure of dII/dVI cluster formation (see Fig 1C, Fig 6C and [24]), could be detected after *in vivo* depletion of rpL17/uL22 or rpL19/eL19 [24,53]. Therefore, the inefficient recruitment of rpL17/uL22 and rpL19/eL19, as well as the weakened pre-ribosomal association of several later acting RBFs belonging to the Nug1 group [41–45,50], provide an explanation why downstream events in LSU maturation, including processing of the ITS2 spacer region of rRNA are impeded upon inhibition of dII/dVI r-protein cluster formation.

We consider the possibility that assembly of the dII/dVI r-protein cluster, besides affecting the association of RBFs and possibly nucleases (see for example levels of a few peptides of the exonuclease Rrp17 in S1 Fig), could have other impacts on early exonucleolytic B1S processing and pre-rRNA stabilization. Inspection of current structure models of the mature yeast LSU suggests that the dII/dVI r-protein cluster might help to protect the LSU rRNA from excessive exonucleolytic digest by hindering the steric accessibility of the 5.8S rRNA 5’ end: dII/dVI cluster r-proteins tightly anchor LSU rRNA domain VI on LSU rRNA domain II through an extensive interaction network and stabilize thereby a lid-like conformation of LSU rRNA domain VI on top of the 5.8S rRNA 5’ end (Fig 5A–5D). In support of the functional relevance of rRNA domain bridging by the dII-dVI cluster all cluster r-proteins analyzed up to now and the ES7 LSU rRNA fragment they are attached to are crucial for early stabilization and efficient exonucleolytic 5’ trimming of LSU pre-rRNA [19,24,26,33]. Moreover, experiments performed in this work indicated that the C-terminal domain of cluster r-protein rpL16, which locks a part of LSU rRNA domain VI, ES39, in a clamp-like fashion into position (Fig 5E–5F), is important for the very same maturation events. Besides, the observation that *in vivo* depletion of the major LSU rRNA domain VI binder rpL3 leads to pronounced destabilization of early LSU precursors underlines the functional significance of the spatial organization of LSU rRNA domain VI for initial steps of LSU maturation (Fig 2, lane 10, [19,24,38]). Consistent with these strong effects, a disturbed folding state of LSU rRNA domain VI in the absence of rpL3 would likely result in increased steric nuclease accessibility of the 5’ end of 5.8S rRNA and of the 3’ end of 25S rRNA, which is embedded in this domain. However, assembly of rpL3 into early LSU precursors seems not to depend on the formation of the dII/dVI r-protein cluster at LSU rRNA domain II (Fig 4). Conclusions about the role of rpL3 for dII/dVI r-protein cluster formation are complicated by the pronounced destabilization of yeast LSU precursors in the absence of rpL3 (Fig 2, [19]).

**Fig 8:**
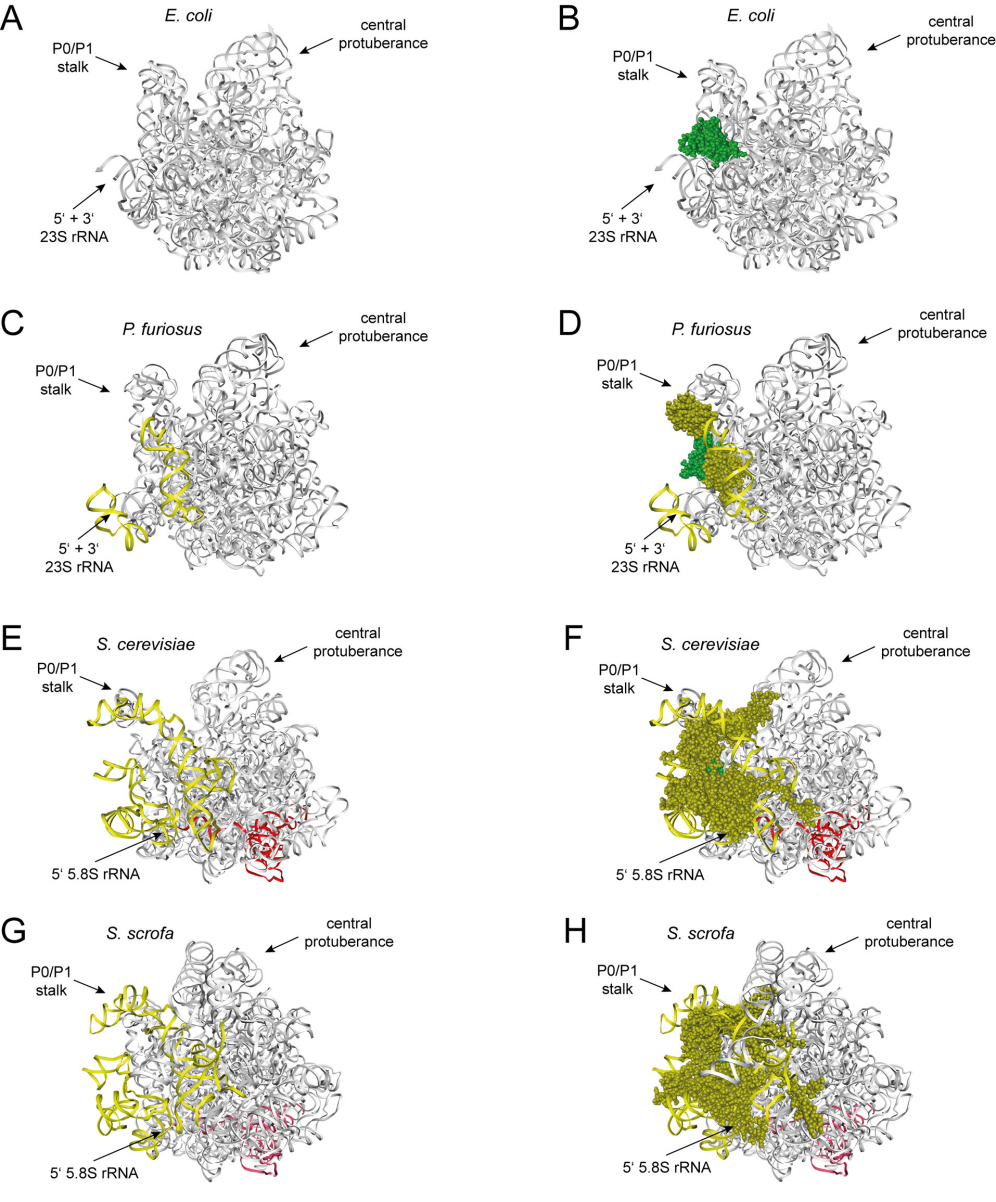
Contacts between LSU rRNA domains II and VI and position of the LSU rRNA 5’ end in bacterial, archaeal and eukaryotic ribosomes. Structure models are shown of the LSU of the bacterium *Escherichia coli* in **A)** and **B)**, the archaeon *Pyrococcus furiosus* in **C)** and **D)**, and the two eukaryotes *Saccharomyces cerevisiae* in **E)** and **F)** and *Sus scrofa* in **G)** and **H)**. LSU rRNA expansion segments ES7 and ES39, and dII/dVI cluster r-proteins are colored in yellow, except the universal conserved globular domain of rpL16/uL13 which is shown in green. 5.8S rRNA is highlighted in red and other parts of LSU rRNA, except ES7 and ES39, are grey. In **A)**, **C)**, **E)** and **G)** only rRNA is shown and in **B)**, **D)**, **F)** and **H)** dII/dVI cluster r-proteins are the only r-proteins visualized.

With exception of the globular N-terminal domain of rpL16/uL13, none of the dII/dVI cluster r-proteins is conserved in bacteria (see discussion in [52] with respect to conservation of rpL6/L6e, rpL14/L14e and rpL16/uL13 in evolution). In line with this, the yeast rRNA expansion segments ES7 and ES39 with their numerous contacts with dII/dVI cluster r-proteins have no counterpart in bacterial LSU rRNA (see the *Escherichia coli* LSU structure in Fig 8A and 8B). At the same time, the spatial arrangement of the LSU rRNA 5’ end seems significantly different in bacteria and eukaryotes. As seen for the *Escherichia coli* LSU, the 5’ end of bacterial 23S rRNA base pairs with the 23S rRNA 3’ end and the resulting helix is rather exposed (Fig 8A and 8B, [54]). Its accessibility is not sterically blocked by LSU rRNA domain VI. With the addition of parts of the expansion segments ES39 and ES7, and the two dII/dVI cluster r-proteins L14e and L33e in some archaeal species the situation is intermediate between the one observed in yeast and *E*. *coli* (Fig 8C and 8D, [55]). Still, archaeal r-protein mediated bridging between LSU rRNA domains II and VI is scarce and the spatial position and base pairing of the LSU rRNA 5’ end largely resembles the one in *E*. *coli* ribosomes. In contrast in eukaryotes, all dII/dVI cluster r-proteins are conserved and current structural models suggest that their general spatial arrangement between LSU rRNA domain II, LSU rRNA domain VI, and the conserved parts of ES7 and ES39 is strikingly similar in yeast and mammalian ribosomes (Fig 8E–8H, [1,56]). Significantly, the number of r-protein mediated contacts between LSU rRNA domains II and VI largely increases from bacteria to eukaryotes and these changes are accompanied by the addition of rRNA elements contacting the relevant r-proteins and by a complete reorganization of ribosomal spatial organization around the LSU rRNA 5’ end. In contrast to bacteria, in eukaryotes the 5.8S rRNA 5’ end base pairs with downstream regions of LSU rRNA domain I, and its position moved to the place, as described above, whose steric accessibility is largely blocked by LSU rRNA domain VI. In terms of functional conservation, the results of knock-down experiments in human cells targeting L13a, the homologue of yeast rpL16/uL13, indicated that its impact on LSU formation is not conserved in mammals [57]. On the other hand, similar experiments with the human homologues of the yeast dII/dVI cluster r-proteins rpL14/L14e and rpL33/L33e (human L14 and L35A) were in agreement with correct formation of the mammalian dII-dVI r-protein cluster being a prerequisite for stabilization and efficient processing of early LSU precursors [58].

In agreement with previous studies, the results of the analyses performed here suggest that formation of the L8/L15 r-protein cluster at LSU rRNA domain I and of the dII/dVI r-protein cluster at LSU rRNA domain II can occur in yeast rather independently of each other (Fig 4, [18]). Still, formation of both clusters is required for initial LSU rRNA precursor maturation steps, including efficient processing at site B1S [19,24,27]. The data presented here underline previous observations indicating that pre-ribosomal association, and therefore possibly recruitment of the large Erb1/Has1 group of LSU biogenesis factors required for these maturation events is affected by L8/L15 r-protein cluster formation (Fig 3, [18]). As discussed previously [18], pre-ribosomal binding sites of several of these factors, including Rlp7, Erb1, Nop7, Cic1 and Nop15 have been detected in close proximity to the ribosomal L8/L15 cluster region and in the ITS2 spacer emanating from there ([28–30], see also S3 Fig for an overview on known binding sites of factors of the Erb1/Has1 group with regard to the ribosomal location of the L8/L15 cluster). In addition, genetic interactions between L8/L15 cluster protein rpL36 and Ebp2 of the Erb1/Has1 group have been observed [27]. Altogether, it seems therefore possible that L8/L15 cluster formation and its impact on the surrounding pre-rRNA fold contribute to a local binding platform for many proteins of the Erb1/Has1 group. As previously suggested, formation of the L8/L15 cluster might promote correct folding of LSU rRNA domain I and thereby base pairing at the 5.8S 5’ end possibly slowing down exonuclease activities [24,59]. The action of 5’ 3’ exonucleases on LSU precursors might be further restricted by the establishment of direct interactions of the 5.8S rRNA 5’ end region with r-proteins assembling downstream of the L8/L15 cluster r-proteins and of several of the Erb1/Has1 group of biogenesis factors [9,28,48,60,61]. Thus, we suggest that r-protein assembly pathways in the three LSU rRNA domains I, II and VI can promote early LSU rRNA stabilization and productive exonucleolytic trimming of the 5.8S rRNA 5’ end by different means. These include specific effects on the pre-ribosomal association of subsets of early acting RBFs, establishment of direct interactions at the LSU rRNA 5’ end, and LSU rRNA domain II and VI mediated hindrance of its steric accessibility. Extensive coupling of partly independent r-protein assembly pathways to early LSU rRNA stabilization should promote the production of ribosomes of appropriate composition and folding states.

## Materials and Methods

### Yeast strains & microbiological procedures

Oligonucleotides, plasmids and yeast strains used in this work are listed in S1, S2 and S3 Tables respectively. The yeast strains expressing chromosomally-encoded TAP-tagged Noc2 were created according to [16,62]. Primers used for amplification of the *TAP-URA3*-cassette from plasmid pBS1539 are listed in S1 Table. Plasmids coding for full length or truncated variants of rpL16 were created as described in S2 Table.

### Affinity purification of (pre-) rRNPs associated with Noc2-TAP using IgG coupled magnetic beads followed by semi-quantitative mass spectrometry

Yeast strains conditionally expressing LSU r-protein genes were cultivated at 30°C in YPG (1% yeast extract, 2% bacto peptone, 2% galactose); expression of the respective genes was shut down by incubating cells in YPD (1% yeast extract, 2% bacto peptone, 2% glucose) for indicated times at 30°C. Affinity purifications of Noc2-TAP and associated pre-ribosomal particles from extracts of the respective yeast strains shifted to glucose containing medium were performed as described in [16]. The eluate fractions of these affinity purifications were further processed for semi quantitative mass spectrometric protein analyses as described in [63].

### Affinity purification of (pre-) rRNPs using anti-FLAG antibody coupled sepharose beads

Affinity purification of (pre-) rRNPs containing tagged variants of rpL16 was performed using anti FLAG antibody coupled sepharose beads as described in [64] with the following modifications. The cell pellet corresponding to 250 ml yeast culture with OD600 = 0.8–1.0 was resuspended in 500 μl cold A200 buffer (20 mM Tris–HCl pH 8, 200 mM KCl, 5 mM MgOAc, 0.2% Triton X-100, 1 mM DTT, 2 mM Benzamidine, 1 mM PMSF) containing 0.04 U/μl RNasin (Promega). A cell lysate was prepared by vigorous shaking of the cell suspension with 1.4 ml glass beads (0.75–1 mm diameter) in a IKA-Vibrax VXR shaker for 15 min, followed by 2 minutes incubation on ice. This procedure was repeated two times. The cell lysate was cleared from cell debris by two centrifugation steps, 1×5 min at 14000 rpm and 1×10 min at 14000 rpm. The protein concentration of the cleared lysate was determined using the Bradford assay. Equal amounts of cell lysate (typically 0.5 ml with 20–50 mg of total protein) were incubated with 100 μl of equilibrated (3× washing with A200 buffer) anti-FLAG M2 beads slurry (Sigma) and rotated for 1.5 h at 4°C. The beads were washed 7 times (1×1 ml, 5×2 ml and 1×10 ml) with cold A200 buffer in a 10 ml column (BioRad).

### RNA extraction, northern blotting and primer extension reactions

RNA was extracted by hot acidic phenol–chloroform treatment as described previously [24]. Northern blotting analyses after RNA separation on formaldehyde/MOPS agarose gel (18S/25S rRNA and their precursors) or Urea/TBE/Polyacrylamide gels (7S pre-rRNA) were done following the protocol published in [65]. Hybridization with the radioactively labelled probes listed in S1 Table was performed as described in [16]. The oligonucleotide O210 (see S1 Table) complementary to a region immediately upstream of the C2 cleavage site in ITS2 was used to detect 5′-ends of 27S and 7S pre-rRNA intermediates by primer extension following the procedure described in [66].

### Data visualization and hierarchical clustering analyses of semi quantitative proteome data of different (pre-) ribosomal particles purified via Noc2-TAP

Hierarchical clustering analyses of semi quantitative mass spectrometry data sets derived from several experiments were done as described in [16,63], using cluster 3.0 software [67]. All observed normalized iTRAQ ratios were expressed in log2 scale. Levels of bait protein Noc2-TAP were used for normalization of semi-quantitative mass spectrometry data. Filtering of the identified proteins of interest was done as described in the respective figure legends. The distance matrices of the data (shown as dendrograms) in Figs 3 and 4 were calculated by the “City block distance” method and hierarchical clustering analyses were done with the “centroid linkage” algorithm. Cluster visualization (tree and heat map) was done with Java Tree view (see http://www.eisenlab.org/eisen/?page_id=42). Mass spectrometry datasets and additional information can be found in S1 File.

### Analysis and visualisation of ribosomal structure models

Ribosomal structure models were obtained from the Research Collaboratory for Structural Bioinformatics Protein Data Bank (RCSB-PDB, http://www.rcsb.org/pdb/home/home.do). Structure files used were 4V4Q for the Escherichia coli LSU [54], 4V6U for the Pyrococcus furiosus LSU [55], 4V88 for the Saccharomyces cerevisiae LSU [1] and 3J7R for the Sus scrofa LSU [56]. They were analysed and visualized with the Discovery Studio 3.5 and 4.1 Visualizer software packages.

## Supporting Information

S1 Fig Experimental dataset: LSU-Factors in Noc2p Fractions. The semi quantitative mass spectrometry results of the 17 individual (biological) replicates of pre-ribosmal particles purified via Noc2-TAP from cells depleted of different LSU r-proteins (shown in Fig 3B) are depicted here in more detail. Changes in levels of individual LSU biogenesis factors in Noc2-TAP fractions from mutant versus wild type cells (as determined by iTRAQ) are depicted as heatmaps (see legend on the left side). All data were normalized to the bait protein Noc2-TAP (iTRAQ ratio was set to 1 for Noc2). The average number of identified peptides for each protein is given in parentheses. Proteins that were only identified with one peptide (in average) are highlighted by an asterisk. In addition to the identified LSU biogenesis factors, average values for the identified housekeeping proteins, SSU r-proteins, LSU r-proteins, and SSU processome components are shown. This dataset was used for the clustering analyses shown in Fig 3B.
(PDF)

S2 Fig Experimental dataset: LSU r-proteins in Noc2p Fractions. The semi quantitative mass spectrometry results of the 17 individual (biological) replicates of pre-ribosomal particles purified via Noc2-TAP from cells depleted of different LSU r-proteins (shown in Fig 4) are depicted here in more detail. Changes in levels of individual LSU r-proteins in Noc2-TAP fractions from mutant versus wild type cells (as determined by iTRAQ) are depicted as heatmaps (see legend on the left side). All data were normalized to the bait protein Noc2-TAP (iTRAQ ratio was set to 1 for Noc2). The average number of identified peptides for each protein is given in parentheses. Proteins that were only identified with one peptide (in average) are highlighted by an asterisk. In addition to the identified LSU r-proteins, average values for the identified housekeeping proteins, SSU r-proteins, LSU biogenesis factors, and SSU processome components are shown. This dataset was used for the clustering analyses shown in Fig 4.
(PDF)

S3 Fig Known ribosomal binding sites of factors of the Erb1/Has1 group. The yeast LSU is shown viewed from the solvent exposed side (left panel) and the subunit interface side (right panel). 5.8S rRNA is colored in blue, other LSU rRNA in white and known binding sites of Erb1/Has1 group factors are highlighted in yellow. The sites where ITS2 pre-rRNA sequences originate from the 5.8S rRNA 3’ end and the 25S rRNA 3’ end are indicated. L8/L15 cluster r-proteins rpL8/eL8, rpL13/eL13, rpL15/eL15 and rpL36/eL36 are shown in brown, dII/dVI cluster r-proteins rpL16/uL13, rpL33/eL33, rpL6/eL6, rpL20/eL20 and rpL14/eL14 in dark green and other LSU rRNA domain II binding r-proteins analyzed in this study (rpL4/uL4, rpL7/uL30, rpL18/eL18, rpL32/eL32) in light green.
(PDF)

S1 File Mass spectrometry datasets and additional information. (ZIP)

S1 Table Oligonucleotides used in this study. (PDF)

S2 Table Plasmids used in this study. (PDF)

S3 Table Yeast strains used in this study. (PDF)
