# Supplementary figures and images for: Studies on the Coordination of Ribosomal Protein Assembly Events Involved in Processing and Stabilization of Yeast Early Large Ribosomal Subunit Precursors
Source: PLoS One. 2015 Dec 7;10(12):e0143768. doi: 10.1371/journal.pone.0143768 (PMC4671574; doi:10.1371/journal.pone.0143768)

depleted  
r-protein

rpL4

rpL7

rpL8

rpL16

rpL18

rpL20

rpL32

rpL33

log2 of  
iTRAQ  
ratio

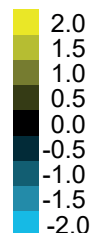

not  
identified

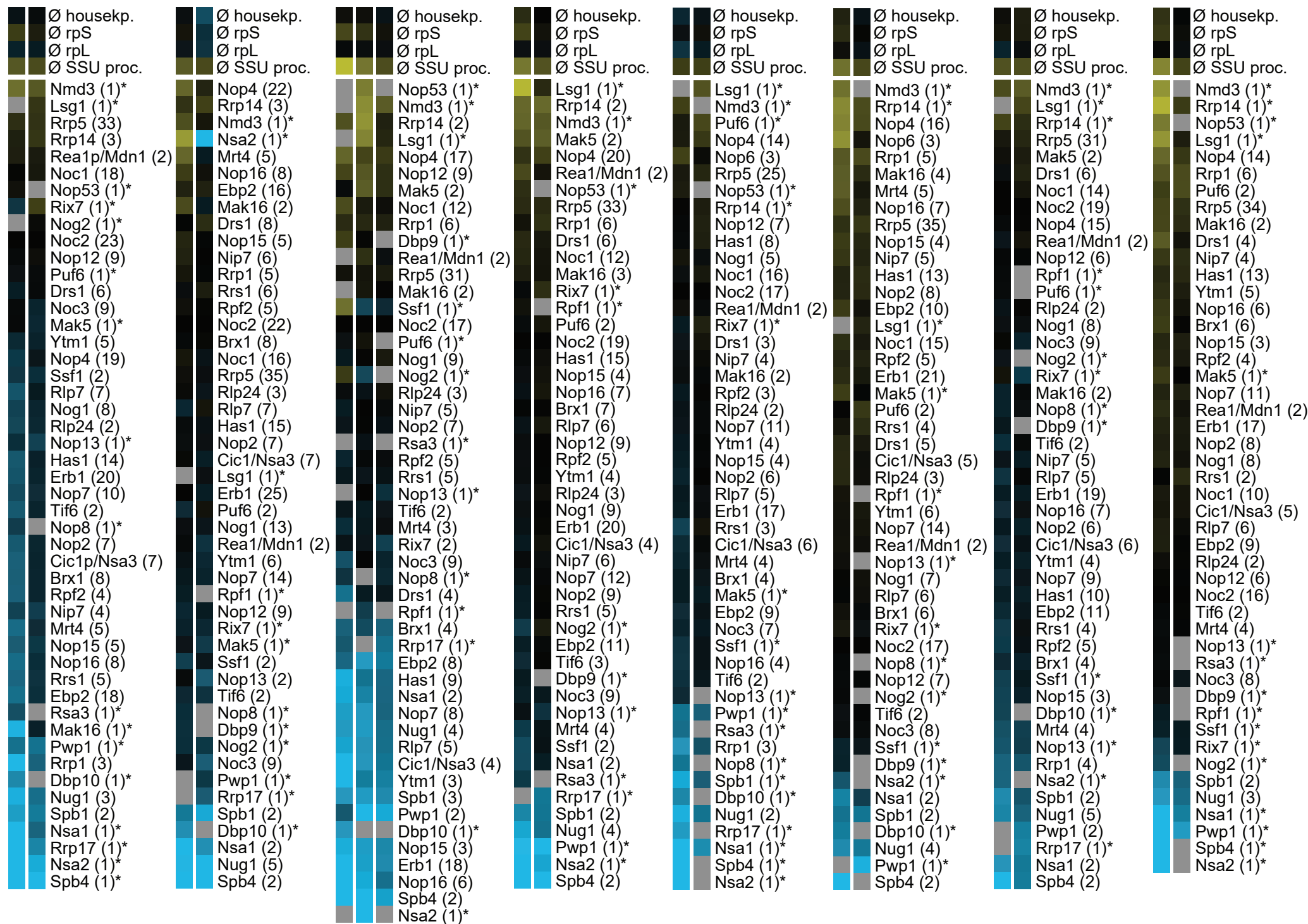

Supplement: S1 Fig — The semi quantitative mass spectrometry results of the 17 individual (biological) replicates of pre-ribosmal particles purified via Noc2-TAP from cells depleted of different LSU r-proteins (shown in Fig 3B) are depicted here in more detail. Changes in levels of individual LSU biogenesis factors in Noc2-TAP fractions from mutant versus wild type cells (as determined by iTRAQ) are depicted as heatmaps (see legend on the left side). All data were normalized to the bait protein Noc2-TAP (iTRAQ ratio was set to 1 for Noc2). The average number of identified peptides for each protein is given in parentheses. Proteins that were only identified with one peptide (in average) are highlighted by an asterisk. In addition to the identified LSU biogenesis factors, average values for the identified housekeeping proteins, SSU r-proteins, LSU r-proteins, and SSU processome components are shown. This dataset was used for the clustering analyses shown in Fig 3B. (PDF) [file pone.0143768.s001.pdf]

depleted r-protein

rpL4

rpL7

rpL8

rpL16

rpL18

rpL20

rpL32

rpL33

log2 of  
iTRAQ  
ratio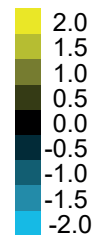not  
identified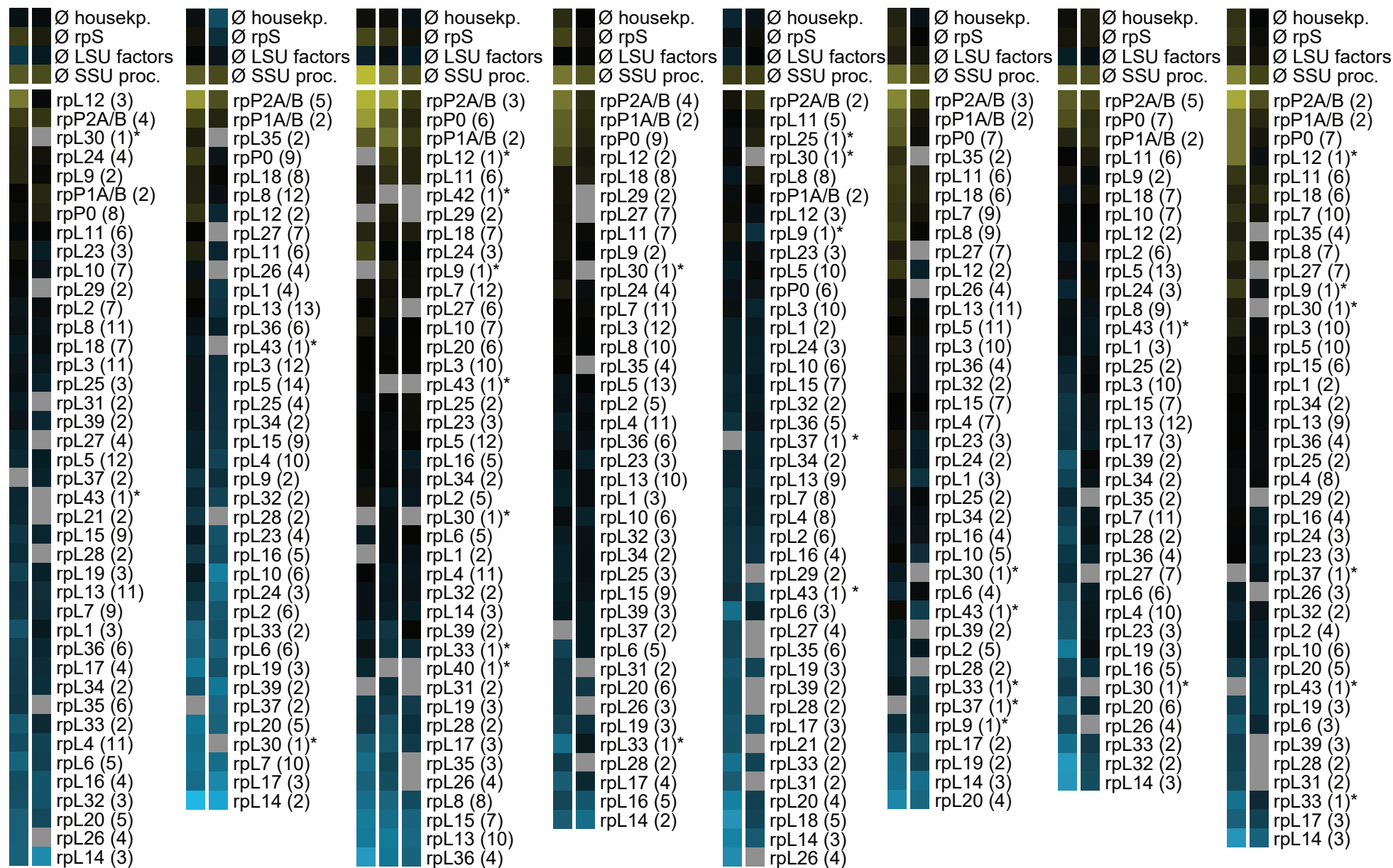

Supplement: S2 Fig — The semi quantitative mass spectrometry results of the 17 individual (biological) replicates of pre-ribosomal particles purified via Noc2-TAP from cells depleted of different LSU r-proteins (shown in Fig 4) are depicted here in more detail. Changes in levels of individual LSU r-proteins in Noc2-TAP fractions from mutant versus wild type cells (as determined by iTRAQ) are depicted as heatmaps (see legend on the left side). All data were normalized to the bait protein Noc2-TAP (iTRAQ ratio was set to 1 for Noc2). The average number of identified peptides for each protein is given in parentheses. Proteins that were only identified with one peptide (in average) are highlighted by an asterisk. In addition to the identified LSU r-proteins, average values for the identified housekeeping proteins, SSU r-proteins, LSU biogenesis factors, and SSU processome components are shown. This dataset was used for the clustering analyses shown in Fig 4. (PDF) [file pone.0143768.s002.pdf]

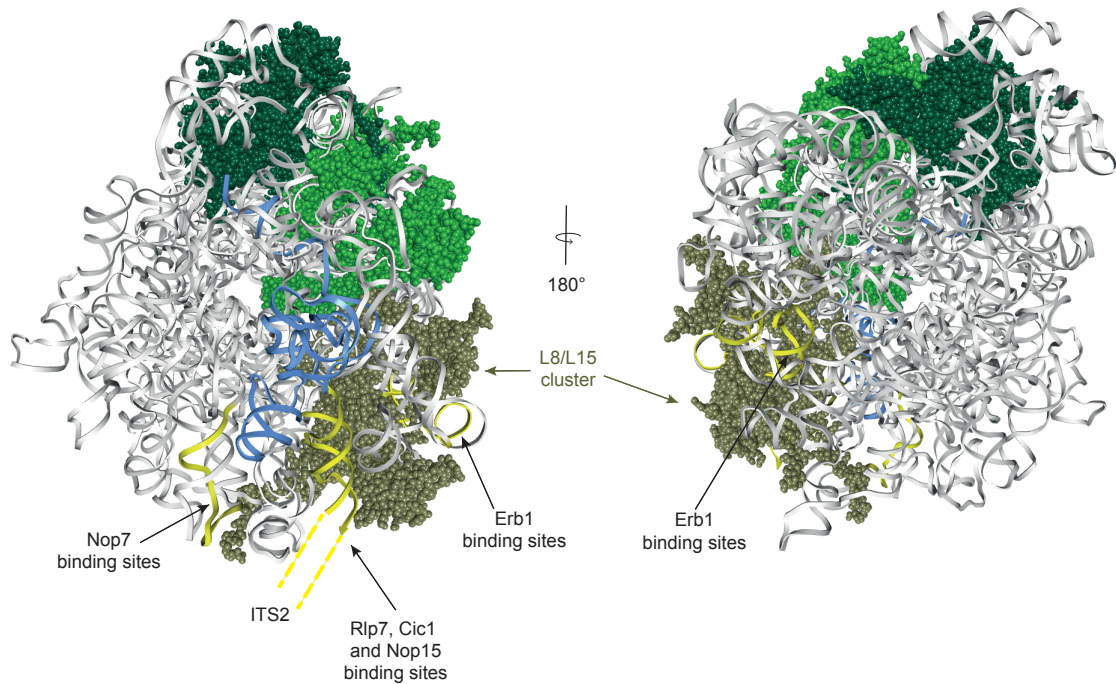

Supplement: S3 Fig — The yeast LSU is shown viewed from the solvent exposed side (left panel) and the subunit interface side (right panel). 5.8S rRNA is colored in blue, other LSU rRNA in white and known binding sites of Erb1/Has1 group factors are highlighted in yellow. The sites where ITS2 pre-rRNA sequences originate from the 5.8S rRNA 3’ end and the 25S rRNA 3’ end are indicated. L8/L15 cluster r-proteins rpL8/eL8, rpL13/eL13, rpL15/eL15 and rpL36/eL36 are shown in brown, dII/dVI cluster r-proteins rpL16/uL13, rpL33/eL33, rpL6/eL6, rpL20/eL20 and rpL14/eL14 in dark green and other LSU rRNA domain II binding r-proteins analyzed in this study (rpL4/uL4, rpL7/uL30, rpL18/eL18, rpL32/eL32) in light green. (PDF) [file pone.0143768.s003.pdf]
