# Supplementary material for: Studies on the Coordination of Ribosomal Protein Assembly Events Involved in Processing and Stabilization of Yeast Early Large Ribosomal Subunit Precursors
Source: PLoS One. 2015 Dec 7;10(12):e0143768. doi: 10.1371/journal.pone.0143768 (PMC4671574; doi:10.1371/journal.pone.0143768)
Supplement: S1 Table — (PDF) [file pone.0143768.s005.pdf]

Primer	Name	Sequence (5´ to 3´ direction)
205	*18S probe*	CATGGCTTAATCTTTGAGAC
207	*A2/A3 probe*	TGTTACCTCTGGGCCC
210	*E/C2 probe*	GGCCAGCAATTTCAAGTTA
212	*25S probe*	CTCCGCTTATTGATATGC
621	*NOC2-TAP fw*	AAGTGATGATGACAACGAAGATGTTGAAATGTCAGACGCT TCCATGGAAAAGAGAAG
622	*NOC2-TAP rev*	CTATTGAATTCAAGACAAAAAATCAAATCTTGCTGAGTTGTACGACTCACTATAG GG
AG1	*18S probe*	AGCCATTCGAGTTTCACTG
AG9	*RPL16B-SpeI*	TTTTTTACTAGTTTCAATCCGTACACTATACAC
O1235	*RPL16B-PstI*	TTTTTTCTGCAGGAATTACTTGACCGTTACTGC
AG10	*RPL16BΔInt*	TTTTTTCCTTAGCATCAATAACAACGACTGGTT
AG11	*RPL16BΔ51-F*	GTCTACTTCTGTTGGTTGGTAATACGAAGATGTTGTTGC
AG12	*RPL16BΔ51-R*	GCAACAACATCTTCGTATTACCAACCAACAGAAGTAGAC
AG13	*RPL16BΔ28-F*	CGCCGAATATTACGCCAAGTAGAGAGCTTTCACCAAAAAGG
AG14	*RPL16BΔ28-R*	CCTTTTTGGTGAAAGCTCTCTACTTGGCGTAATATTCGGCC
AG15	*RPL16B-BamHI*	TTTTTTGGATCCATGTCTCAACCAGTCGTTGTTATT
AG16	*RPL16B-PstI-orf*	TTTTTTCTGCAGGTAACCGAAAGAAGCCAATTG
AG17	*RPL16BΔ51-PstI*	TTTTTTCTGCAGCCAACCAACAGAAGTAGACAACT
AG18	*RPL16BΔ28-PstI*	TTTTTTCTGCAGCTTGGCGTAATATTCGGCG
