# Supplementary material for: Studies on the Coordination of Ribosomal Protein Assembly Events Involved in Processing and Stabilization of Yeast Early Large Ribosomal Subunit Precursors
Source: PLoS One. 2015 Dec 7;10(12):e0143768. doi: 10.1371/journal.pone.0143768 (PMC4671574; doi:10.1371/journal.pone.0143768)
Supplement: S2 Table — (PDF) [file pone.0143768.s006.pdf]

| | | | |
|---|---|---|---|
| pAGH1 | Ycplac33-RPL16B | *URA3* | product of a PCR with genomic DNA as template and primers AG9/O1235 was SpeI/PstI digested and cloned into Ycplac33 |
| pAGH2 | Ycplac33-RPL16BΔInt | *URA3* | product of a PCR with pAGH1 as template and primers AG9/AG10 was SpeI/Bpu10i digested and cloned into SpeI/Bpu10i digested pAGH1 |
| pAGH3 | Ycplac33-*rpl16bΔ51* | *URA3* | Site-directed mutagenesis using QuickChangeII Site-Directed Mutagenesis kit from Agilent with the oligos AG11/AG12 and pAGH2 as template. * product of a PCR with mutagenized pAGH3 as template and primers AG9/O1235 was subcloned into Ycplac33 to avoid other mutations in the vector backbone |
| pAGH4 | Ycplac33-*rpl16bΔ28* | *URA3* | Site-directed mutagenesis using QuickChangeII Site-Directed Mutagenesis kit from Agilent with the oligos AG13/AG14 and pAGH2 as template. * product of a PCR with mutagenized pAGH4 as template and primers AG9/O1235 was subcloned into Ycplac33 to avoid other mutations in the vector backbone |
| Yeplac195 | Yeplac195 | *URA3* | Gietz and Sugino, Gene 74 (2), p. 527-534 |
| TK487 | Yeplac195-pRPS28-RPS24-FLAG | *URA3* | Ferreira-Cerca et al., 2007 (suppl. data) |
| pAGH5 | Yeplac195-pRPS28-RPL16B-FLAG | *URA3* | product of a PCR with pAGH2 as template and primers AG15/AG16 was BamHI/PstI cloned into TK487 |
| pAGH6 | Yeplac195-pRPS28-*rpl16bΔ51* -FLAG | *URA3* | product of a PCR with pAGH3 as template and primers AG15/AG17 was BamHI/PstI cloned into TK487 |
| pAGH7 | Yeplac195-pRPS28-*rpl16bΔ28* -FLAG | *URA3* | product of a PCR with pAGH4 as template and primers AG15/AG18 was BamHI/PstI cloned into TK487 |
| pAGH8 | Ycplac33-pRPS28-RPL16B-FLAG | *URA3* | pvuII cassette from pAGH5 was cloned into Ycplac33 |
| pAGH9 | Ycplac33-pRPS28-*rpl16bΔ51* -FLAG | *URA3* | pvuII cassette from pAGH6 was cloned into Ycplac33 |
| pAGH10 | Ycplac33-pRPS28-*rpl16bΔ28* -FLAG | *URA3* | pvuII cassette from pAGH7 was cloned into Ycplac33 |
