# Supplementary material for: Studies on the Coordination of Ribosomal Protein Assembly Events Involved in Processing and Stabilization of Yeast Early Large Ribosomal Subunit Precursors
Source: PLoS One. 2015 Dec 7;10(12):e0143768. doi: 10.1371/journal.pone.0143768 (PMC4671574; doi:10.1371/journal.pone.0143768)
Supplement: S3 Table — (PDF) [file pone.0143768.s007.pdf]

JW8423	pGAL-RPL7	-	*ura3-52, trp1-101, lys2-801, his3-d200, leu2-d1, rpl7b::KANMX6, GAL-3HA-RPL7A (TRP1)*	Pöll et al., 2009
JW8425	pGAL-RPL18	-	*ura3-52, trp1-101, lys2-801, his3-d200, leu2-d1, rpl18b::KANMX6, GAL-3HA-RPL18A (TRP1)*	Pöll et al., 2009
Y1768	pGAL-RPL3 NOC2-TAP	TK822	*his3-1, leu2-0, ura3-0, YOR063w::kanMX4, noc2::NOC2-TAP-URA3*	strain Y966 was transformed with a PCR product on pBS1539 with primers 621/622
Y1769	pGAL-RPL8 NOC2-TAP	TK882	*his3-1, leu2-0, ura3-0, met15-0, lys2-0, YHL033c::HIS3MX6, YLL045c::kanMX4, noc2::NOC2-TAP-URA3*	strain Y1097 was transformed with a PCR product on pBS1539 with primers 621/622
Y1770	pGAL-RPL16 NOC2-TAP	TK810	*his3-1, leu2-0, ura3-0, YNL069c::KANMX4, YIL133c::HIS3MX6, noc2::NOC2-TAP-URA3*	strain Y931 was transformed with a PCR product on pBS1539 with primers 621/622
Y1771	pGAL-RPL20 NOC2-TAP	TK821	*his3-1, leu2-0, ura3-0, YOR312c::HIS3, YMR242c::KANMX4, noc2::NOC2-TAP-URA3*	strain Y932 was transformed with a PCR product on pBS1539 with primers 621/622
Y1772	pGAL-RPL32 NOC2-TAP	TK880	*his3-1, leu2-0, ura3-0, YBL092w::KANMX4, noc2::NOC2-TAP-URA3*	strain Y1102 was transformed with a PCR product on pBS1539 with primers 621/622
Y1773	pGAL-RPL33 NOC2-TAP	TK813	*his3-1, leu2-0, ura3-0, YPL143w::kanMX4, YOR234c::HIS3MX6, noc2::NOC2-TAP-*	strain Y1096 was transformed with a PCR product on pBS1539 with primers 621/622
Y1879	BY4742 NOC2-TAP	-	*his3-1, leu2-0, ura3-0, lys2-0, noc2::NOC2-TAP-URA3*	strain Y207 was transformed with a PCR product on pBS1539 with primers 621/622 (Pöll et al., 2009)
Y2786	pGAL-RPL18 NOC2-TAP	-	*ura3-52, trp1-101, lys2-801, his3-d200, leu2-d1, rpl18b::KANMX6, GAL-3HA-RPL18A (TRP1), noc2::NOC2-TAP-URA3*	strain JW8425 was transformed with a PCR product on pBS1539 with primers 621/622
Y2788	pGAL-RPL4 NOC2-TAP	-	*ura3-52, trp1-101, lys2-801, his3-d200, leu2-d1, rpl4b::KANMX6, GAL-3HA-RPL4A (TRP1), noc2::NOC2-TAP-URA3*	strain JW8402 was transformed with a PCR product on pBS1539 with primers 621/622
Y2790	pGAL-RPL7 NOC2-TAP	-	*ura3-52, trp1-101, lys2-801, his3-d200, leu2-d1, rpl7b::KANMX6, GAL-3HA-RPL7A (TRP1), noc2::NOC2-TAP-URA3*	strain JW8423 was transformed with a PCR product on pBS1539 with primers 621/622
Hm653	pGAL-RPL16 RPL16BΔInt-FLAG	TK810 pAGH5	*his3-1, leu2-0, ura3-0, YNL069c::KANMX4, YIL133c::HIS3MX6*	strain Y931 was transformed with plasmid pAGH5
Hm654	pGAL-RPL16 *rpl16bΔ51*-FLAG	TK810 pAGH6	*his3-1, leu2-0, ura3-0, YNL069c::KANMX4, YIL133c::HIS3MX6*	strain Y931 was transformed with plasmid pAGH6
Hm655	pGAL-RPL16 *rpl16bΔ28*-FLAG	TK810 pAGH7	*his3-1, leu2-0, ura3-0, YNL069c::KANMX4, YIL133c::HIS3MX6*	strain Y931 was transformed with plasmid pAGH7
